# Mitochondrial dysfunction in heart failure and its therapeutic implications

**DOI:** 10.3389/fcvm.2022.945142

**Published:** 2022-08-24

**Authors:** Miaosen Liu, Jialan Lv, Zhicheng Pan, Dongfei Wang, Liding Zhao, Xiaogang Guo

**Affiliations:** ^1^Clinical Medicine, Zhejiang University School of Medicine, Hangzhou, China; ^2^Department of Cardiology, The First Affiliated Hospital, Zhejiang University School of Medicine, Hangzhou, China

**Keywords:** heart failure, mitochondria, calcium, reactive oxygen species, mitophagy, fusion and fission

## Abstract

The ATP consumption in heart is very intensive to support muscle contraction and relaxation. Mitochondrion is the power plant of the cell. Mitochondrial dysfunction has long been believed as the primary mechanism responsible for the inability of energy generation and utilization in heart failure. In addition, emerging evidence has demonstrated that mitochondrial dysfunction also contributes to calcium dysregulation, oxidative stress, proteotoxic insults and cardiomyocyte death. These elements interact with each other to form a vicious circle in failing heart. The role of mitochondrial dysfunction in the pathogenesis of heart failure has attracted increasing attention. The complex signaling of mitochondrial quality control provides multiple targets for maintaining mitochondrial function. Design of therapeutic strategies targeting mitochondrial dysfunction holds promise for the prevention and treatment of heart failure.

## Introduction

Mitochondrion is cellular organelle surrounded by two membranes, which is the source of energy production within the cells. Beyond its critical role in supplying energy, mitochondrion is also closely related to reactive oxygen species (ROS) production, Ca^2+^ homeostasis and related signal transduction (1, 2). Mitochondrion is highly plastic, and the number, shape and function remain relatively stable under normal conditions. Mitochondrial homeostasis is crucial for cell fate (3–5).

Mitochondrion is able to quickly adapt to changing conditions. Mitochondrion adjusts its morphology and function to meet the needs of cell, which is conducive to respond to internal and external stimuli and maintain the physiological function (3). Conversely, mitochondrial dysfunction may in turn cause cell fate transition, leading to the damage of structure, function and metabolism, and even cell death (6, 7), which takes part in the genesis and development of a variety of diseases (8–11). Accumulating studies demonstrated that mitochondrial dysfunction contributes to the pathogenesis of common cardiovascular diseases (CVD) (12–16), culminating in end-stage heart failure (17, 18). In this review, we mainly discuss mitochondrial dysfunction-mediated energy metabolism disturbance, Ca^2+^ dysregulation, oxidative stress, proteostasis imbalance, and mitophagy deficiency in the development of heart failure.

## Mitochondrial quality control

Mitochondrial quality control mainly includes the ubiquitin-proteasome system (UPS) which recognizes and degrades misfolded and damaged proteins to maintain mitochondrial protein homeostasis (19), mitochondrial fusion and fission to ensure the number, morphology and proper intracellular distribution of mitochondria (20), and mitophagy to selectively remove damaged and redundant mitochondria (21). Mitochondrial quality control is of vital importance to maintain mitochondrial homeostasis. Mitochondrial metabolic disorders, morphological and functional changes are closely related to the occurrence, development and treatment of various diseases such as aging, cancer, metabolic disorders, neurodegenerative diseases and CVD (22–24).

## Disturbance of mitochondrial energy metabolism in heart failure

Heart failure is a clinical syndrome resulted from a wide range of causes, including ischemic, hypertensive, inflammatory, and toxic heart diseases. The heart is a very high energy consumption organ and must continually generate ATP to support muscle contraction and relaxation. The energy substrates in mitochondria of failing heart are switched. There is about 70% of ATP coming from the beta-oxidation using fatty acids as the primary fuel in mitochondria of healthy adult heart, and the contribution of fatty acids beta-oxidation to overall ATP production can even reach to almost 100% of the total energy requirement of the heart (25). Glucose, lactate, pyruvate, ketone body and amino acid are also substrates for myocardial energy provision (26). During the pathological progression of heart failure, the energy metabolism mode of cardiomyocytes gradually changes, with an increased proportion from glucose and a decreased proportion from fatty acids beta-oxidation (27). This metabolic remodeling of substrate utilization shifts cardiac metabolism to a fetal energy metabolism (28, 29). Compared with fatty acids, the oxygen consumption of energy production is lessened when glucose is used as the substrate (30). To some extent, this switch helps to maintain cardiac function in the process of chronic heart disease.

The oxidative phosphorylation in mitochondria is impaired and energy starvation is observed in the human biopsies of failing heart (31). Heart failure is mainly divided into ischemic and non-ischemic heart failure. Ischemic heart failure is related to coronary artery diseases especially myocardial infarction and accounts for about 50% of heart failure (32). The energy substrates adaptively shift toward glucose, which increases the stoichiometric ratio of ATP production to oxygen consumption and improves cardiac function in ischemic heart (33, 34). Thus replacing fatty acids by glucose is considered to be able to increase oxygen efficiency and benefit energy provision in failing heart. The “adaptive” mechanism during the progression of heart failure is not well understood. Amorim et al. reported that insulin-induced phosphorylation of Akt is normal and the expression of glucose transporter type 4 is unchanged, while the expression of the genes regulating fatty acid oxidation, e.g., long-chain-acyl-coenzyme A dehydrogenase, carnitine palmitoyltransferase 1 (CPT I) and peroxisome proliferator-activated receptor-α, is reduced in the infarcted hearts (35). Oxidative phosphorylation is badly damaged due to severe hypoxia in heart with acute myocardial infarction. Anaerobic glycolysis is inefficient to produce adequate ATP to meet the energy requirement of the heart and also results in the accumulation of lactate (36, 37). ATP depletion and acidosis lead to impaired myocardial contractility and injured membrane pumps and ion channels. The alterations of membrane pumps and ion channels cause mitochondrial swelling, Ca^2+^ accumulation and the opening of mitochondrial permeability transition pore (mPTP), which is prominent in cardiomyocyte of myocardial ischemic injury and has been demonstrated to play a critical role in various forms of cell death in myocardial ischemia/reperfusion injury (38, 39).

Non-ischemic heart failure is mainly related to hypertrophic cardiomyopathy and dilated cardiomyopathy (DCM) (34). Mitochondrial energy substrates switch is prevalent in non-ischemic heart failure, which is driven by energy metabolic reprogramming leading to altered enzymes and substrate flux (29). Accumulating evidence has indicated that enzymes involved in fatty acids beta-oxidation pathway decrease significantly in the myocardium from heart failure patients. Martin et al. reported that total CPT and CPT II activities decrease in failing heart, and carnitine deficiency is related to ventricle dysfunction (40). Very long-chain acyl-CoA dehydrogenase (VLCAD) deficiency is the most common defect of mitochondrial long-chain fatty acid β-oxidation. Mitochondrial energy metabolism is impaired in VLCAD^–/–^ mice, and hypertrophic cardiomyopathy is a typical manifestation of VLCAD deficiency in human (41, 42). The changes in energy metabolic gene expression and substrates progressively lead to energy starvation. On the other hand, the pathological remodeling increases energy consumption in diseased heart. The impairment of mitochondrial energy metabolism leads to energy deficiency, which is in contradiction with the increased energy demand in hypertrophic heart caused by pathological remodeling.

Recent studies revealed that mitochondrial pyruvate carrier (MPC), which transports pyruvate into the mitochondria, is reduced in failing human and mouse hearts (43, 44). Cardiac assist device was found to increase MPC expression in the myocardium and promote myocardial recovery in patients with chronic heart failure (45). Mice with cardiac-specific deletion of MPC1 or/and MPC2 resulted in cardiac hypertrophy, dilated cardiomyopathy, and contractile dysfunction (43–46). These results indicate an important role of pyruvate metabolism in myocardial metabolism and function. Pyruvate dehydrogenase (PDH), an enzyme converts pyruvate into acetyl-CoA, was reported to be inactivated in advanced pathological conditions of heart failure (47, 48). However, a recent study found that PDH is activated at an early phase before the down-regulation of fatty acid oxidation and tricarboxylic acid (TCA) cycle, suggesting that PDH activation is one of the earliest events to compensate for metabolic impairment from myocardial damage (49). Increased PDH expression and activity were evident with decreased expression of PDH kinase 4, MPC1 and MPC2, sustaining the capacity for PDH to facilitate glucose metabolism in end-stage systolic heart failure (50).

## Dysregulation of Ca^2+^ homeostasis in heart failure

As a second messenger, Ca^2+^ plays a central role in myocardial excitation-contraction coupling (51). Using the energy of ATP hydrolysis, Ca^2+^ pump transports Ca^2+^ ions from the cytoplasm into the sarcoplasmic reticulum (SR) or out of the cell. Therefore, energy deficiency always couples with the dysregulation of Ca^2+^ transportation in failing heart, which consequently leads to excitation-contraction uncoupling and cardiac dysfunction (52). Dysregulation of Ca^2+^ homeostasis is also associated with abnormal leak of Ca^2+^ from SR through ryanodine receptors (RyR) and results in increased cytosolic Ca^2+^ at baseline but reduced cytosolic Ca^2+^ transients during excitation (53, 54). Overwhelming studies revealed that Ca^2+^ leaked from the SR *via* RyR2 causes mitochondrial Ca^2+^ overload, which plays a key role in heart failure (55).

Mitochondrial Ca^2+^ uptake and efflux also influence cytosolic Ca^2+^ homeostasis (56). Mitochondrial Ca^2+^ uptake depends on the mitochondrial Ca^2+^ uniporter (MCU) (57, 58), while mitochondrial Ca^2+^ efflux is mainly regulated by the mitochondrial Na^+^/Ca^2+^/Li^+^ exchanger (56). Mitochondria can sense the change of cytosol free Ca^2+^ and maintain intracellular Ca^2+^ homeostasis by regulating the opening and closing of MCU and Na^+^/Ca^2+^/Li^+^ exchanger, so as to prevent cytosolic Ca^2+^ overload and cell damage (56). However, excessive mitochondrial Ca^2+^ uptake causes mitochondrial Ca^2+^ overload, which impairs mitochondrial function, leading to decreased ATP generation and increased mitochondrial ROS (mtROS) production, and is a key determinant in heart failure (55). MCU is also a mitochondrial redox sensor and MCU oxidation enhances its channel activity leading to mitochondrial Ca^2+^ overload, increased mtROS, and even cell death (59). In addition, increased intracellular Ca^2+^ leads to the activation of calpain, which mediates mitochondrial damage in diseased hearts (60). Recent studies revealed that the activation of calpain results in mitochondrial damage and subsequent cardiac dysfunction by impairing mitophagy and promoting mitochondrial fission and apoptosis (13, 61).

On the other hand, if the level of mitochondrial Ca^2+^ falls below a critical threshold level, Ca^2+^-dependent activation of tricarboxylic acid (TCA) cycle enzymes is disrupted, and consequently ATP generation is impaired whereas NADH oxidation is increased. This pathological change was demonstrated in both myocytes isolated from failing heart (62) and in an animal model of heart failure (63). Therefore, mitochondrial Ca^2+^ homeostasis is critical for normal heart function.

## Accumulation of mitochondrial reactive oxygen species in heart failure

Mitochondria are one of the main sources of ROS production within the cell. TCA cycle-dependent electron transfer complexes are distributed in the inner membrane of mitochondria. Most electron transfer process is coupled with the generation of ATP, and only 1–2% of the electrons are transferred to produce superoxide anion, which can be scavenged by superoxide dismutase (64). The mtROS is mainly generated from the complex I and complex III in the electron transport chain (65). The basal level of mtROS can fulfill essential physiological functions by acting as signaling molecule. It plays an important physiological role in mediating gene expression, regulating cell cycle and cell differentiation, and is of great significance for stress response, cell survival, cell proliferation, etc. (1).

However, excessive accumulation of mtROS plays an important role in pathological process of heart failure. Dai et al. demonstrated that mtROS contributes to angiotensin II-induced cardiac hypertrophy and heart failure (66). Chouchani et al. showed that mtROS generation during the early stages of reperfusion promotes myocardial ischemia/reperfusion injury (67). It is recognized that mtROS drives acute cardiovascular events such as electrical instability and chronic proteome remodeling in heart failure (68). Dysfunction of the mitochondrial respiratory chain is accompanied by increased generation of mtROS, leading to oxidative stress and resulting in mitochondrial protein and DNA damage, membrane lipid peroxidation, and the opening of the mPTP. The opening of the mPTP causes the release of cytochrome C and cell apoptosis, consequently promotes the progression of heart failure (69). It is well believed that apoptosis is mainly responsible for the cumulative loss of cardiomyocyte in failing heart, and causally contributes to myocardial dysfunction progression (70). Recent study also demonstrated that mtROS induced NLRP3 inflammasome activation and cardiomyocyte pyroptosis in DCM, uncovering a novel event in the initiation and progression of heart failure (71).

Nicotinamide adenine dinucleotide phosphate (NADPH) oxidase (NOX) is one of the predominant sources of ROS in the heart. Accumulating data indicate that complex crosstalk and interaction exist between NOX and mtROS (72, 73). Under conditions of oxidative stress, NOX-derived ROS can cause lipid peroxidation of mitochondrial membrane and the opening of redox-sensitive mitochondrial ATP-sensitive K^+^ channel (mitoKATP), leading to mtROS generation from the electron transport chain (73).

## Dysregulation of mitochondrial proteostasis in heart failure

Mitochondria have their own genome encoding mitochondrial specific proteins. The mitochondrial proteome is composed of 1,000∼1,500 proteins, encoded by both mitochondrial and nuclear genomes (74, 75). Mitochondrial proteostasis is essential for the function of cell. Normal mitochondrial function requires coordinated gene expression in the nucleus and in mitochondria (19). The UPS controls protein transport across the mitochondrial outer membrane, and primarily degrades outer membrane proteins that are improperly imported or damaged/mislocalized (19). Furthermore, mitochondrial chaperones and proteases govern protein folding and degrade damaged proteins inside mitochondria. Dysregulation of mitochondrial proteostasis leads to proteotoxic insults and eventually cell death (19).

Under cellular stress conditions, both mitochondrial-encoded and nuclear-encoded proteins are misfolded and dysfunctional. The generation of mtROS compromises protein integrity and folding. The proteotoxic stress within mitochondria activates mitochondrial unfolded protein response (UPRmt), which induces the transcription of mitochondrial chaperones (e.g., heat shock protein 60), antioxidants (e.g., thioredoxin 2) and proteases (e.g., caseinolytic mitochondrial matrix peptidase proteolytic subunit, CLPP; YME1 like 1 ATPase, YME1L; OMA1 zinc metallopeptidase, OMA1) (76). UPRmt re-establish mitochondrial proteostasis by facilitating protein folding or repairing misfolded proteins, and degrading unrepairable proteins (75).

UPRmt is of great benefit to the preservation of ATP production, reduction of mtROS accumulation, and inhibition of mitochondria-mediated cell death, and showed a protective effect in chronic and acute cardiac injury (77). Activating transcription factor associated with stress-1 (ATFS-1), an UPRmt-inducing transcription factor, was showed to preserve ATP production by promoting the assembly and function of oxidative phosphorylation components during mitochondrial stress (78). Upregulation of mitochondrial Lon protease (LONP1), a component of UPRmt, protected the myocardium from cardiac stress and limited ischemia/reperfusion injury (79). Recent study found that intensive sympatho-excitation leads to pathological cardiac hypertrophy and fibrosis, which is coupled with decreased UPRmt and increased mitochondrial proteotoxic stress (80). These myocardial morphological alterations is closely associated with heart failure with preserved ejection fraction (HFpEF) (81). Smyrnias et al. demonstrated that UPRmt is activated during chronic pressure overload and pharmacological enhancement of the UPRmt alleviates mitochondrial and contractile dysfunction in the stressed heart (82). Xu et al. also found that choline attenuated the mito-nuclear protein imbalance and activated UPRmt to preserve the ultrastructure and function of mitochondria in hypertrophic heart (83). Further studies confirmed that pharmacological UPRmt activation exerts cardioprotective effect in an ATF5-dependent manner in mouse models of ischemia-reperfusion injury and transverse aortic constriction-induced cardiac hypertrophy (84, 85). All these indicate that UPRmt may play an important protective role in stressed heart and may be potential therapeutic target for heart failure.

Reciprocal regulation between mitochondrial proteases YME1L and OMA1 is critical in UPRmt. They antagonistically regulate mitochondrial proteostasis by ATP-dependent network. When mitochondrial depolarization with preserved ATP levels, YME1L is further activated and OMA1 is degraded through a mechanism involving YME1L. Whereas in the absence of ATP, OMA1 is activated and stabilized by membrane depolarization, and subsequently promotes YME1L degradation (86, 87). It was reported that pressure overload decreases YME1L expression and cardiac-specific overexpression of YME1L improves cardiac function in pressure overload-induced heart failure (88). Acin-Perez demonstrated that OMA1 ablation averts cardiomyocyte death in three different mouse models of heart failure: tachycardiomyopathy, heart failure with preserved left ventricular ejection fraction, and left ventricular myocardial ischemia and hypertrophy (89). All these suggested that the regulation of YME1L and OMA1 is potential target for preventing the progression of heart failure associated with distinct types of etiologies.

However, there is contradictory evidence that UPRmt may be associated with adverse events within the heart. The release of mitochondrial chaperone molecule heat shock protein 60 was observed to promote proinflammatory tumor necrosis factor-α, which correlated with increased myocyte apoptosis in heart failure (90). The mitochondrial matrix protease CLPP plays a central role in the activation of the UPRmt, deletion of CLPP in heart increased the synthesis of oxidative phosphorylation subunits and attenuated the mitochondrial cardiomyopathy (91). Cao et al. showed that parvostatin improved left ventricular function and slowed the progression of heart failure in mice by blocking UPRmt activator c-Jun (92). The contradictory effects of UPRmt on heart may due to its intensive degree. A moderate activation of UPRmt may maintain normal mitochondrial and cardiac function by removing/repairing damaged mitochondrial proteins, while an excessive UPRmt may exacerbate mitochondrial dysfunction and cardiac dysfunction due to a massive cleavage of mitochondrial proteins (77).

Moreover, hyperacetylation of mitochondrial proteins has been found in the myocardium of patients and animal model with heart failure (93). The hyperacetylation of TCA cycle enzymes and electron transfer enzymes results in decreased activity of these enzymes, leading to disruption of mitochondrial bioenergetics and redox homeostasis (93, 94). Reduced fatty acid beta-oxidation is associated with increased short-chain acyl-CoA in the failing heart, indicating an imbalance in the utilization and supply of acetyl-CoA may lead to increased acetylation of mitochondrial proteins (17). Sirtuin 3 (SIRT3), a key deacetylase in mitochondria, has been demonstrated to be downregulated in the failing heart, and SIRT3 knockout mice are susceptible to develop transverse aortic constriction-induced heart failure (95). This result suggests that reduced protein deacetylation may be also responsible for protein hyperacetylation in mitochondria of the failing heart.

## Imbalance of mitochondrial dynamics in heart failure

Mitochondrial dynamics including fusion and fission determines the number, morphology and distribution of mitochondria, and modulates mitochondrial functions to respond properly to body demands (20). Imbalance of mitochondrial dynamics disturbs energy and mtROS generation, Ca^2+^ homeostasis and proteostasis, even induces cell death in the heart (96). Mitofusin 1 (MFN1) and MFN2 are the core components of the mitochondrial fusion machinery and coordinately regulate mitochondrial fusion (97). Mitochondrial fission requires fission protein dynamin-related protein 1 (Drp1) (98).

The balance between fusion and fission ensures the number and morphology of mitochondria in cardiomyocytes. In addition, mitochondria are tightly packed between the sarcomere myofibrils or closely localized to the SR in order to provide crosstalk between sarcomeres and efficient SR-mitochondria during excitation–contraction coupling (99). Downregulation of mitochondrial dynamics regulator MFN2 has been found resulting in mitochondrial fragmentation and contributing to the development of heart failure in rats and in patients with pulmonary arterial hypertension (PAH) (100). Decreased MFN2 expression and excessive mitochondrial fission were also observed in diabetic mice and promoted the development of diabetic cardiomyopathy, indicating mitochondrial dynamics is therapeutic target for intervention in diabetic cardiomyopathy (101).

Activation of fission protein Drp1 and aberrant mitochondrial fission were observed in PAH, and the Drp1 inhibitor Mdivi-1 attenuated mitochondrial fragmentation and improved exercise capacity, right ventricular function, and hemodynamics in experimental PAH (102). Recent study showed that lipid overload induced Drp1 acetylation and eventually resulted in cardiomyocyte death and heart dysfunction (103). Phosphorylated activation of Drp1 and mitochondrial fission contributed to cardiomyocyte pyroptosis in non-ischemic DCM mice, and culminating in end-stage heart failure (71).

## Impairment of mitophagy in heart failure

Mitophagy is a selective autophagic process that eliminates dysfunctional mitochondria. It is essential for mitochondrial quality control and cell function (21). Mitophagic deficiency results in the accumulation of dysfunctional/damaged mitochondria, which reduces the capacity of ATP production and increases the generation of mtROS (21). Continuous constitutive autophagy has a crucial role in maintaining cardiac structure and function. The phosphatase and tensin homolog (PTEN)-induced putative kinase 1 (PINK1)/Parkin pathway is an important pathway in regulating mitophagy (104). Mitophagy is impaired in aged and doxorubicin-treated hearts, and inhibition of Parkin-mediated mitophagy subsequently promotes cardiac dysfunction and overexpression of Parkin attenuates the functional decline in mouse hearts (105). Overexpression of Parkin also protects cardiac myocytes against hypoxia-mediated cell death and Parkin protein deficiency aggravates myocardial damage and reduces survival following myocardial infarction (106). In addition, AMP-activated protein kinase α2 (AMPKα2) prevents the progression of heart failure by promoting mitophagy *via* PINK1 phosphorylation (107).

FUN14 domain-containing protein 1 (FUNDC1) is an outer mitochondrial membrane protein and serves as a receptor to mediate mitophagy (104). It was reported that hypoxic preconditioning induces FUNDC1-dependent mitophagy, regulates mitochondrial homeostasis, and protects the heart from ischemia/reperfusion injury (108). Autophagy protein 5 (Atg5) deficiency decreases mitophagy, leading to increased ROS production and NF-κB activity, thereby contributing to cardiac inflammation and injury (109, 110). Wang et al. found that mitophagy coordinates the UPRmt to attenuate sepsis-mediated myocardial injury, and endogenous UPRmt is a downstream signal of mitophagy to maintain mitochondrial homeostasis in the case of mitophagy inhibition (111). Insufficient mitophagy has been associated with multiple forms of cardiomyopathy, including age-related cardiomyopathy (112), obesity-associated cardiomyopathy (113) and diabetic cardiomyopathy (114). Moreover, pharmacological or genetic inhibition of mitophagy often exacerbates the progression of heart failure in multiple animal models of cardiovascular diseases (104, 115).

## Targeting mitochondrial dysfunction as a therapeutic strategy

Classic treatments recommended in patients with heart failure include diuresis to reduce cardiac preload, vasodilation to reduce the pressure load, inhibition of angiotensin II to block the pathological remodeling, Digoxin or inotropes to enhance myocardial contractility, and so on (116). Although recent evidence showed cardiac myosin activator Omecamtiv Mecarbil (117) and the soluble guanylate cyclase stimulator Vericiguat (118) can benefit patients with heart failure, the outcome of heart failure especially HFpEF is still difficult to change fundamentally.

**FIGURE 1:**
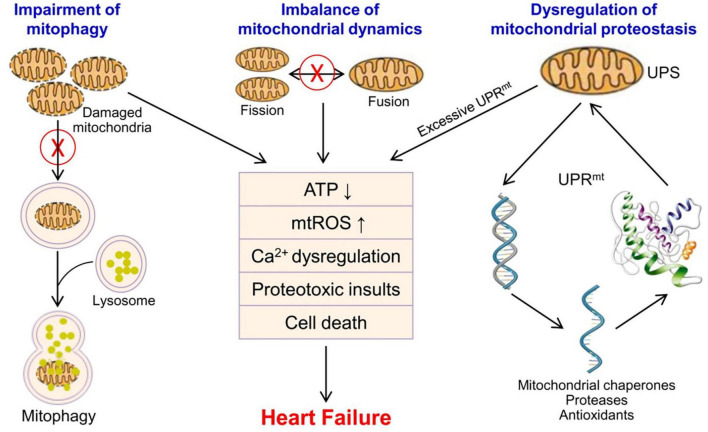
Mitochondrial quality control and mitochondrial dysfunction in the pathophysiology of heart failure. Mitochondrial quality control mainly includes UPS/UPRmt, mitochondrial fusion and fission, and mitophagy to ensure the number, morphology and function of mitochondria. Mitochondrial dysfunction leads to energy metabolism disturbance, Ca^2+^ dysregulation, oxidative stress, proteotoxic insults, and cardiomyocyte death in the heart, and contributes to the progression of heart failure. mtROS, mitochondrial reactive oxygen species; UPS, ubiquitin-proteasome system; UPRmt, mitochondrial unfolded protein response.

Mitochondrial dysfunction results in energy metabolism disturbance, Ca^2+^ dysregulation, oxidative stress and proteotoxic insults, and leads to a final outcome-cardiomyocyte death in the heart (Figure 1). The loss of cardiomyocyte contributes to reduced ventricular systolic dysfunction, and is of central importance in the development of heart failure (71, 119, 120). Mitochondrial dysfunction-related cell death may be potential therapeutic targets for heart failure. Several of microRNAs have been shown to inhibit mitochondrion-mediated apoptosis and improve cardiac function by regulating mitochondrial fission and fusion (121–123). Targeting mitochondrial dysfunction-related pyroptosis is also showed to improve cardiac function in DCM mice (71). However, these preliminary findings mainly derived from *in vitro* and preclinical animal models, and lots of work need to be done to further evaluate their potential of cardioprotection in human.

It was reported that the overexpression of catalase targeted to mitochondria but not wild type catalase in peroxisomes ameliorates cardiac hypertrophy and diastolic dysfunction in mice (66). Mitochondria-targeted antioxidant MitoQ was found to decrease heart dysfunction after myocardial ischemia-reperfusion (124), and attenuate hypertension-induced cardiac hypertrophy (125). Recent study also showed that mitoTEMPOL, an mtROS scavenger, attenuates nicotine-induced myocardial remodeling and cardiac dysfunction (126). Coenzyme Q10, an electron carrier in mitochondria, is thought to reduce oxidative stress because of its antioxidant activity. Al Saadi et al. included 11 studies to review the efficacy of coenzyme Q10 in heart failure, and concluded that the included studies provide moderate-quality evidence for the benefit of coenzyme Q10 in reducing all-cause mortality and hospitalization related to heart failure. However, there was low-quality evidence to conclude whether coenzyme Q10 improves either left ventricular ejection or exercise capacity (127). Large-scale and high quality clinical trials are needed to further evaluate the efficacy of coenzyme Q10. Due to the essential role of mtROS in cell function and survival, a precise control between ROS production and detoxification is a decisive issue need to be solved.

Recent study showed that the administration of sodium-glucose co-transporter 2 (SGLT2) inhibitor Empagliflozin significantly reduced cardiovascular death and hospitalization for heart failure in patients regardless of the presence or absence of diabetes (128). It is speculated that SGLT2 inhibitor might improve mitochondrial energetics in the heart by offering β-hydroxybutyrate as an attractive substrate for oxidation and protect against heart failure (129). Empagliflozin was also proved to reduce cytosolic Na^+^ and increase mitochondrial Ca^2+^ in cardiomyocytes independent of SGLT inhibition, probably by inhibition of Na^+^/H^+^ exchanger (130). Further evaluation is needed to elucidate whether this effect contributes to the beneficial effect of Empagliflozin on heart failure.

CGP-37157, a selective inhibitor of the mitochondrial Na^+^/Ca^2+^ exchanger, was shown to maintain mitochondrial Ca^2+^ and ameliorate pathological myocardial remodeling, left ventricular dysfunction and arrhythmias (63). By targeting mitochondrial electron transfer chain, a novel strategy normalizing NAD^+^/NADH redox balance was showed to improve myocardial energetics and cardiac function in failing mouse heart (131). NAD^+^ precursors may have the potential and worth to evaluate their effect on heart failure (132). Moreover, Perhexiline has been reported to improve cardiac energetics and symptom status in heart failure patients (133, 134). It is speculated that the modification of myocardial energy substrate by inhibiting CPT is the primary mechanism underlying the benefit of Perhexiline. However, this hypothesis is not supported by all available evidence. Pleiotropic or unanticipated mechanisms may be relevant to the action of Perhexiline in heart failure (135, 136).

Accumulating evidence identified that MPC abundance mediated pathological cardiac hypertrophy and overexpression of MPC protected against cardiac hypertrophy and dysfunction (44, 45), highlighting the potential of MPC in preventing cardiac remodeling and heart failure.

## Conclusion

Mitochondrial dysfunction is a well-known hallmark of heart diseases, and strongly linked to the pathological development of cardiac dysfunction (17). The complicated signaling of mitochondrial quality control and related cell death offers diverse targets for inhibiting mitochondrial dysfunction. Design of therapeutic strategies targeting the signal molecules in mitochondrial dysfunction holds promise for the prevention and treatment of heart failure (Table 1).

**TABLE 1:** Therapeutic strategies targeting mitochondria for treatment of heart failure.

Principal targets	Drugs/strategies	Diseases/models	Effects	References
Energetics	SGLT2 inhibitor	Heart failure in patients regardless of the presence or absence of diabetes NCT03057977*	Reduce the risk of cardiovascular death or hospitalization for heart failure	(128)
Energetics	Perhexiline (inhibiting CPT-1)	Hypertrophic cardiomyopathy patients NCT00500552*	Corrects energy deficiency and improves exercise capacity	(133)
Energetics	Perhexiline (inhibiting CPT-1)	DCM CT00841139*	Improves cardiac energetics and symptom status	(134)
Energetics	Overexpression of MPC	Transverse aortic constriction-induced heart failure	Increased TCA cycle intermediates, and Protects against cardiac hypertrophy and failure	(44)
Energetics	Overexpression of MPC	Drug-induced hypertrophy	Attenuates cardiac hypertrophy	(45)
NAD^+^/NADH ratio	Elevation of NAD^+^ levels by stimulating the NAD^+^ salvage pathway	Transverse aortic constriction-induced heart failure	Improve myocardial energetics and cardiac function	(131)
NAD^+^/NADH ratio	Nicotinamide mononucleotide (NAD^+^ precursor)	Friedreich’s ataxia cardiomyopathy mouse model	Improves diastolic and normalizes systolic function	(132)
mtROS	Overexpression of catalase targeted to mitochondria	Angiotensin II-induced cardiac hypertrophy and Galphaq overexpression- induced heart failure	Ameliorates cardiac hypertrophy and diastolic dysfunction	(66)
mtROS	mitoTEMPOL	Nicotine-induced myocardial remodeling and cardiac dysfunction	Attenuates nicotine-induced cardiac remodeling and dysfunction	(126)
mtROS	MitoQ	Hypertension-induced cardiac hypertrophy	Attenuates cardiac hypertrophy	(125)
mtROS	MitoQ	Myocardial ischemia-reperfusion injury	Decreases heart dysfunction, cell death, and mitochondrial damage	(124)
mtROS and Energetics	Coenzyme Q10	People with heart failure	Reduce all-cause mortality and hospitalization related to heart failure	(127)
Mitochondrial Ca^2+^ homeostasis	CGP-37157	Aortic constriction combined with daily β-adrenergic receptor stimulation	Ameliorate myocardial remodeling, left ventricular dysfunction and arrhythmias	(63)
Mitochondrial fission	miR-484, miR-361, miR-499	Myocardial infarction	Reduces apoptosis and myocardial infarction	(121–123)
Mitochondrial fission and mtROS	NOX1 and NOX4 inhibitor	DCM	Attenuates pyroptosis and myocardial dysfunction	(71)
Mitophagy	Overexpression of Parkin	Aged heart	Attenuates heart functional decline	(105)
Mitophagy	Urolithin A (an inducer of mitophagy)	Sepsis-mediated myocardial injury	Attenuates sepsis-related myocardial injury	(111)

*Indicated ClinicalTrials.gov number.

## Author contributions

ML prepared the table and wrote the manuscript. JL and ZP drew the figure and revised the manuscript. DW and LZ contributed to the original idea of the review. XG supervised the work and provided research funding support. All authors have read and approved the submitted version.
